# Insect-based diet, a promising nutritional source, modulates gut microbiota composition and SCFAs production in laying hens

**DOI:** 10.1038/s41598-017-16560-6

**Published:** 2017-11-24

**Authors:** Luca Borrelli, Lorena Coretti, Ludovico Dipineto, Fulvia Bovera, Francesca Menna, Lorenzo Chiariotti, Antonio Nizza, Francesca Lembo, Alessandro Fioretti

**Affiliations:** 1 Department of Veterinary Medicine and Animal Productions, University of Naples Federico II, Naples, Italy; 2 Institute of Experimental Endocrinology and Oncology “Gaetano Salvatore” IEOS, National Research Council CNR, Naples, Italy; 3 Department of Advanced Biomedical Science, University of Naples Federico II, Naples, Italy; 4 Department of Molecular Medicine and Medical Biotechnologies, University of Naples Federico II, Naples, Italy; 5 Department of Pharmacy, University of Naples Federico II, Naples, Italy; 6 Task Force on Microbiome Studies, University of Naples Federico II, Naples, Italy

## Abstract

Insects could be potential nutritional sources both for humans and animals. Among these, *Hermetia illucens*, with good amount of chitin and proteins, represents a suitable diet replacement for laying hens. Little is known about insect diet effects on the microbial ecology of the gastrointestinal tract and bacterial metabolites production. In this study we investigated the effect of *H*. *illucens* larvae meal administration on cecal microbiota and short chain fatty acids (SCFAs) production in laying hens. 16S rDNA sequencing showed strong differences between cecal microbiota of soybean (SD) and insect diet (ID) groups both in type and relative abundance (unweighted and weighted beta diversity) of microbial species. In particular, *Bacteroides plebeius*, *Elusimicrobium minutum*, *Alkaliphilus transvaalensis*, *Christensenella minuta*, *Vallitalea guaymasensis* and *Flavonifractor plautii* represented the principal contributors of changes in gut microbiota composition of ID group (FDR p-values < 0.05). Of these, *F*. *plautii*, *C*. *minuta* and *A*. *transvaalensis* have the potential to degrade the chitin’s insect meal and correlated with the observed high levels of gut SCFAs produced in ID group. These microorganisms may thus connect the chitin degradation with high SCFAs production. Our results suggest *H*. *illucens* as a potential prebiotic by well feeding gut microbiota.

## Introduction

Linneus in the 1735 work *Systema Naturae* wrote: “*Larvae assate in deliciis habentur*” [roasted larvae are delicious] describing the larvae of *Rynchophorous* spp.

The global demand for food, especially animal based protein sources, is drastically increasing. Therefore, finding new alternative protein sources is one of the most interesting goals for human and animal nutrition and health. In this case, insects as food and feed seem to be an interesting resource with many environmental and health benefits. About 1900 edible insect species are consumed around the world^1^. Insects are considered part of the natural diet of chickens containing between 30% and 70% of protein on a dry matter basis, fats (about 35%), minerals and vitamins and, for this reason, protein-rich insects are a promising alternative to traditional protein sources, reducing environmental pollution and feed costs^2–4^. Insects are also much more efficient in converting feed to body weight than conventional livestock and research into rearing insects as food and feed on a large scale remains a priority despite the production system being still too expensive. Among the different species, the black soldier fly *Hermetia illucens* (Diptera: Stratiomyidae) seems to be very interesting as a sustainable alternative for food and feed and is considered a good candidate for mass production^1^. *H*. *illucens* is found in abundance and naturally occur around the manure piles of large poultry, pigs and cattle. As a component of a complete diet, *H*. *illucens* prepupae have been found to support good growth in chickens and other animals^3,5–8^. These larvae have natural antibiotics property modifying the microflora of manure, potentially reducing harmful bacteria, such as *Escherichia* coli 0157:H7 and *Salmonella enterica* in hen manure^9,10^. The black soldier fly contain chitin, a naturally occurring polysaccharide considered to be one of the most abundant biopolymers in nature^11^. Chitin is not degraded, absorbed in the small intestine and it can be fermented by the microbiota of the large intestine. Studies of humans and mice suggested that chitin may restore the compositional balance of the microbial community. In addition, chitin, or derivate, seems to exhibit anti-viral, anti-tumor, antifungal activities and antimicrobial properties and a bacteriostatic effect on Gram-negative bacteria, *Escherichia coli*, *Vibrio cholerae*, *Shigella dysenteriae* and *Bacteriodes fragile* ^12^. Insect diet, indeed, may decrease the use of antibiotics in the poultry industry controlling the antimicrobial resistance and its adverse effects on human health^1^.

The gut microbiota plays an important role in its vertebrate host facilitating the digestion of food or feed components^13^, fermenting the diet ingredients to short chain fatty acids (SCFAs), as major products^14^. In chickens, the gut microbiota is still under observation and the predominant microbes in the duodenum, caecum and feces belong to Firmicutes (30–50%) and Bacteroidetes phyla (between 10 and 50%)^15,16^. Previous studies demonstrated that feed additives (prebiotics and probiotics) improve chicken gut functionality and consequentially the health status^15,17^. More recently, Marono *et al*. (2017) observed that *H*. *illucens* larvae administration has good effects on laying hen health status, reducing serum and eggs cholesterol and triglycerides levels^18^. These beneficial effects may be due to the chitin amount provided by diet. We hypothesized in this study that insect-based diet might modulates the gut microbiota and its principal metabolites, improving the health status in laying hens. To this aim, we deep sequenced the gut bacterial community and evaluated the SCFAs concentrations to assess the effect of *H*. *illucens* larvae meal administration in laying hens.

## Results

### Effect of *H*. *illucens* on laying hen health status and eggs quality

After 21 weeks of soybean and insect meal administration (SD and ID groups, respectively), all birds were clinically healthy, neither mortality or diarrhea or sickness signs were observed in the 2 groups throughout the entire experiment, indicating that *H*. *illucens* had no negative effects on laying hen health status as reported in the same experimental design previously published^18^ (Table 1). The insect meal was also able to affect some nutritional characteristics of the eggs (Table S1).

**Table 1:** Feed intake, body weight and serum lipids levels of laying hens fed with insect and soybean meal.

Parameter	SD group	ID group
Feed intake, g/d/hen	125.80 ± 1.96	108.31 ± 3.11***
Body weight, kg	2.09 ± 0.04	1.89 ± 0.04***
Cholesterol, mg/dl	134.56 ± 8.14	108.44 ± 7.17**
Triglycerides, mg/dl	1,941.88 ± 209.00	1,296.63 ± 148.33**

The data are means and standard errors of the measurements after 24 weeks of diet administration. P-values were calculated by two tailed Student’s t-test; **p < 0.01, ***p < 0.001^18^.

### Effect of insect-based diet on gut microbiota structure

Cecal microbiota analyses were performed in order to describe and compare the gut bacterial composition of two groups of laying hens fed with soybean and insect meal-based diet (SD and ID, respectively; n = 6 pool of 2 samples/group); sequencing of V3-V4 regions of 16S rRNA gene from cecal samples generated 178,376 high quality reads assigned to a total of 1,461 operational taxonomic units (OTUs). A sequencing depth of 5,234 sequences/sample, with good’s coverage > 96.9% and clustered in 1,123 OTUs, was considered to elaborate the results and analyze the effect of insect meal-based diet on gut microbial communities with respect to SD.

**Figure 1:**
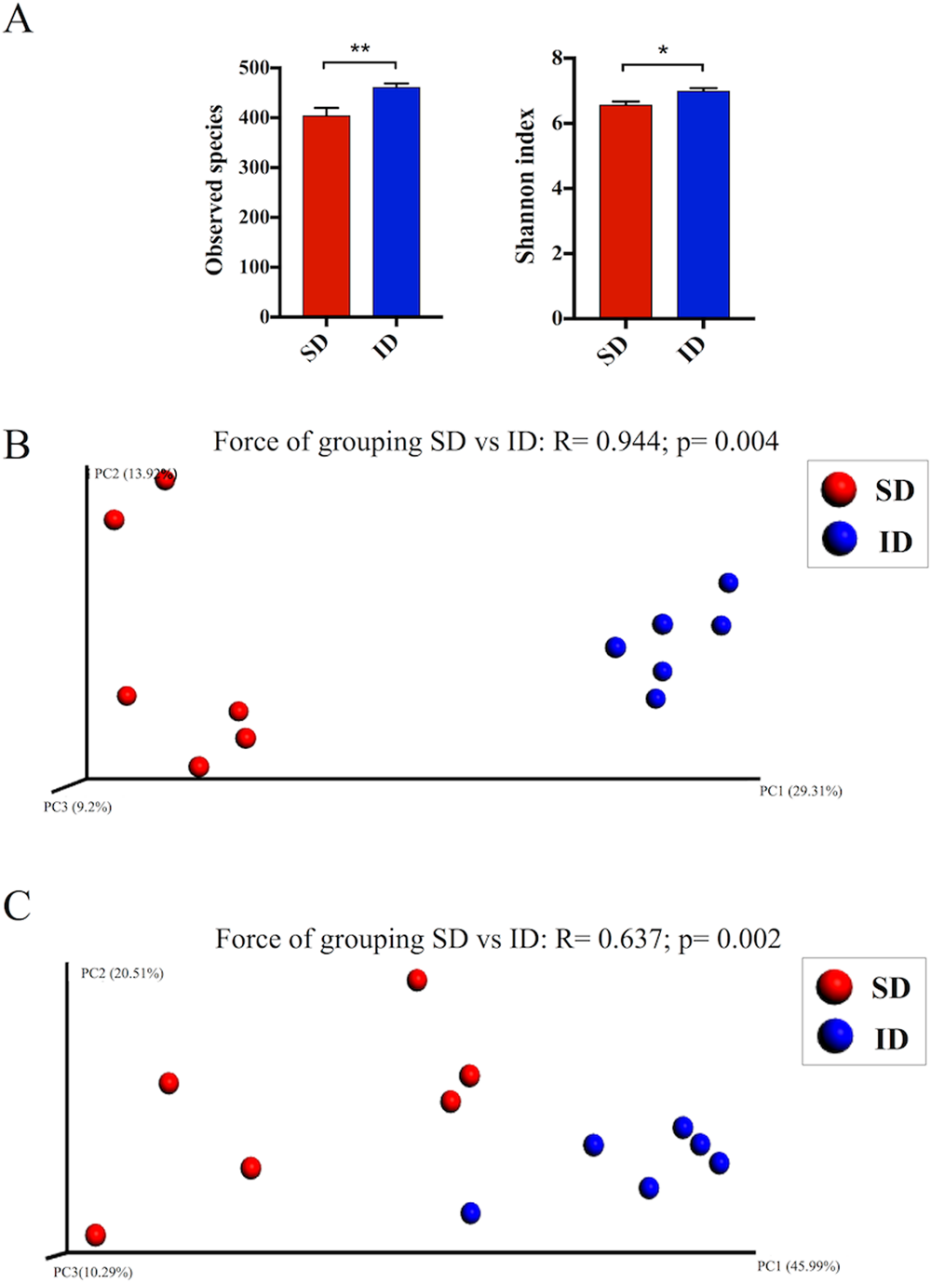
Cecal microbiota structure after insect-based diet administration. (**A**) Diversity within SD and ID microbial communities measured by number of observed species and Shannon index. Data are mean ± SEM. Significant differences are indicated by *p < 0.05 and **p < 0.01 (two-sided Student’s two-sample t-test). (**B**, **C**) PCoA plots based, respectively, on unweighted and weighted UniFrac distances of SD and ID gut microbial communities (5,234 sequences/sample).

Insect meal administration significantly increased the diversity within microbial populations as indicated by a higher number of observed species and Shannon entropy increase in ID samples with respect to SD samples (Fig. 1A). Moreover, strong differences were detected between cecal microbiota of SD and ID groups both in type (unweighted beta diversity) and relative abundance (weighted beta diversity) of microbial species (Fig. 1B and C). In particular, R statistic ANOSIM computed on phylogenetic distances among samples, revealed that ID administration promoted a higher shift in bacterial community assortment (R = 0.944, p = 0.004, Fig. 1B) rather than in abundance of shared species (R = 0.637, p = 0.002; Fig. 1C), as displayed in unweighted unifrac PCoA plot by the net clusterization of ID samples far from SD samples (Fig. 1B).

### Change of gut microbiota composition after insect-based diet

**Figure 2:**
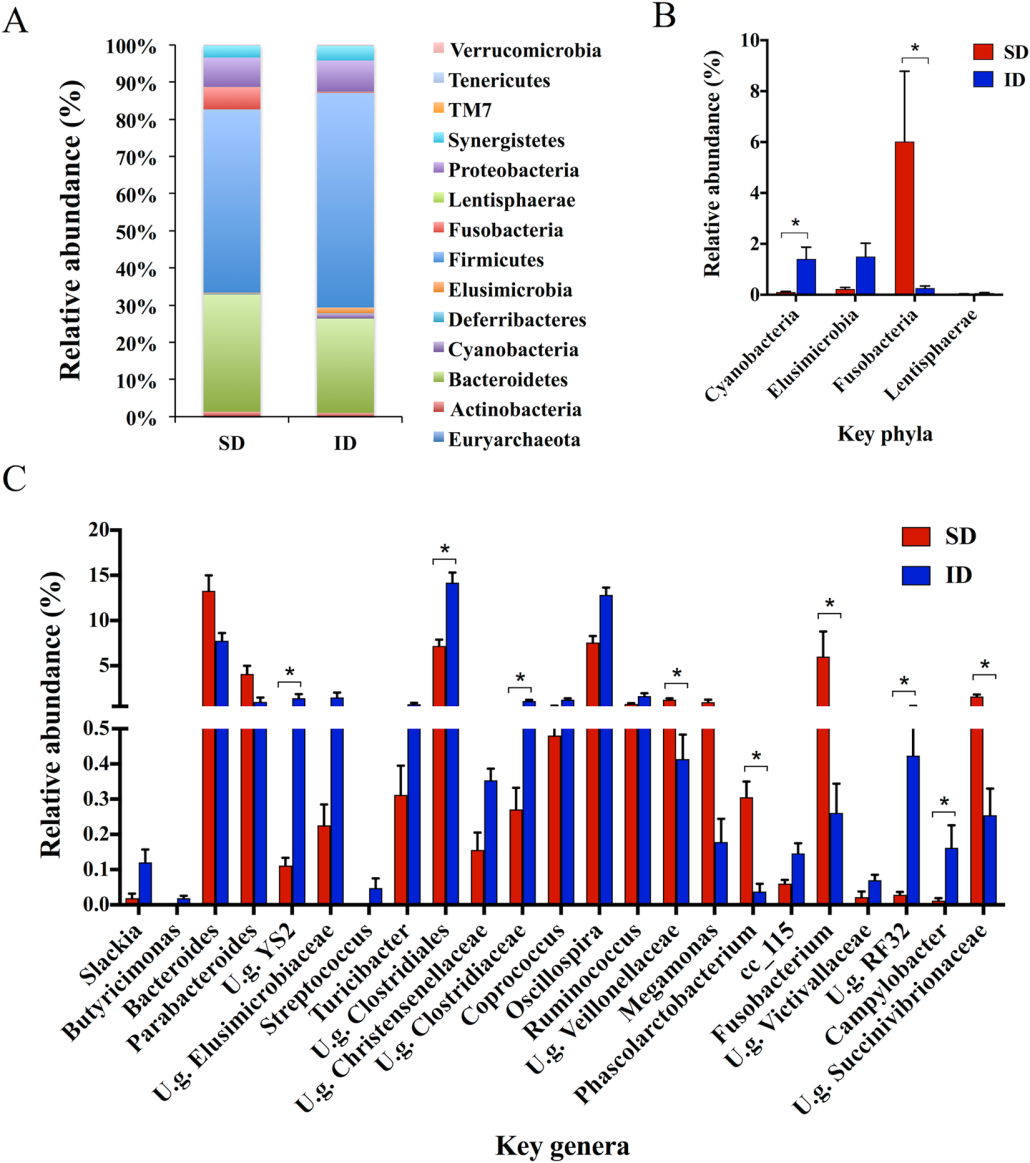
(**A**) Phylogenetic composition at phylum level of SD and ID microbial communities. (**B**,**C**) Relative abundances (%) of bacterial phyla and genera, respectively, found to be significantly different between SD and ID groups (p < 0.05; mean ± SEM). *p < 0.05, **p < 0.01 after FDR correction.

Firmicutes (49.28 ± 3.16% in SD and 57.69 ± 2.37% in ID; mean ± SEM), Bacteroidetes (31.52 ± 3.28 and 25.40 ± 1.30%, in SD and ID, respectively) and Proteobacteria (7.92 ± 1.31% in SD and 8.38 ± 0.47% in ID) were the most abundant bacterial phyla detected in both groups (Fig. 2A) in line with previous studies describing hen cecal microbiota^19^. Insect-based diet in ID group induced a significant increase of Elusimicrobia, Lentisphaerae (p < 0.05) and Cyanobacteria (p < 0.05 after FDR correction) and a decrease of Fusobacteria (p < 0.05 after FDR correction) compared to laying hens fed with a soybean-based diet (Fig. 2B). At genus level, among the 91 bacterial genera detected in all cecal samples, the relative abundance of 23 genera was statistically influenced by the type of diet fed (p < 0.05), with 9/23 genera highly different between the SD and ID groups (p < 0.05 after FDR correction; Fig. 2C). Furthermore, statistical analysis at genus level revealed that 18/23 key genera belonged to Bacteroidetes, Firmicutes and Proteobacteria, although these differences were not observable at phylum level (Fig. 2C). Sequencing analysis of insect and soybean meal, revealed no changes in relative abundances of identified key genera considered as discriminatory between the ID and SD groups (data not shown).

Additionally, we processed the sequencing data using SPINGO’s high-resolution approach^20^, in order to obtain the bacterial species assignment of key genera with significant differences in relative abundance between SD and ID groups (Table 2). Among the species with relative abundance >1%, *Bacteroides plebeius*, *Elusimicrobium minutum*, *Alkaliphilus transvaalensis*, *Christensenella minuta*, *Vallitalea guaymasensis* and *Flavonifractor plautii* were significantly increased after insect meal administration, representing the principal contributors of changes in gut microbiota composition of ID group. Indeed, the bacterial species reduced by ID administration were *Bacteroides salanitronis*, *Parabacteroides merdae*, *Succinatimonas hippei* and unclassified species of genera *Phascolarctobacterium* and *Fusobacterium* (Table 2). Modulation of the relative abundance of the described species upon ID administration is reported in unweighted PCoA biplot (Fig. S1), displaying taxonomic factors driving the clustering of samples.

**Table 2:** SPINGO species classification of key genera identified in SD vs ID comparison.

Key genera	Species	SD (%)	ID (%)
*Bacteroides*	*Bacteroides coprocola*	2.97 ± 1.01	1.20 ± 0.25
	*Bacteroides heparinolyticus*	2.47 ± 0.85	1.38 ± 0.19
	*Bacteroides plebeius*	1.00 ± 0.24	3.00 ± 0.58*
	*Bacteroides salanitronis*	4.37 ± 0.58	0.45 ± 0.15**
*Parabacteroides*	*Parabacteroides merdae*	3.69 ± 0.80	0.82 ± 0.51*
*Elusimicrobiaceae*	*Elusimicrobium minutum*	0.23 ± 0.06	1.51 ± 0.52*
*Clostridiales*	*Alkaliphilus crotonatoxidans*	1.74 ± 0.34	3.04 ± 0.77
	*Alkaliphilus transvaalensis*	0.12 ± 0.04	1.25 ± 0.32**
	*Christensenella minuta*	0.66 ± 0.10	3.39 ± 0.35**
	*Vallitalea guaymasensis*	0.40 ± 0.06	1.03 ± 0.17*
*Oscillospira*	*Flavonifractor plautii*	1.36 ± 0.16	4.61 ± 0.71**
	*Intestinimonas butyriciproducens*	3.13 ± 0.29	4.61 ± 0.61
	*Oscillibacter valericigenes*	2.61 ± 0.41	2.70 ± 0.09
*Ruminococcus*	*Clostridium leptum*	0.53 ± 0.04	1.06 ± 0.20
*Veillonellaceae*	*Unclussified sp*	1.25 ± 0.15	0.41 ± 0.07**
*Fusobacterium*	*Unclussified sp*	6.02 ± 2.77	0.26 ± 0.08**
*Succinivibrionaceae*	*Succinatimonas hippei*	1.61 ± 0.21	0.25 ± 0.08**

Only species with relative abundance >1% are reported (mean ± SEM; n = 6/group). Variations in species’ relative abundances between SD and ID were assessed using nonparametric Kruskal-Wallis test taking into account False Discovery Rate (FDR) corrected p-values; *p < 0.05, **p < 0.01.

**Figure 3:**
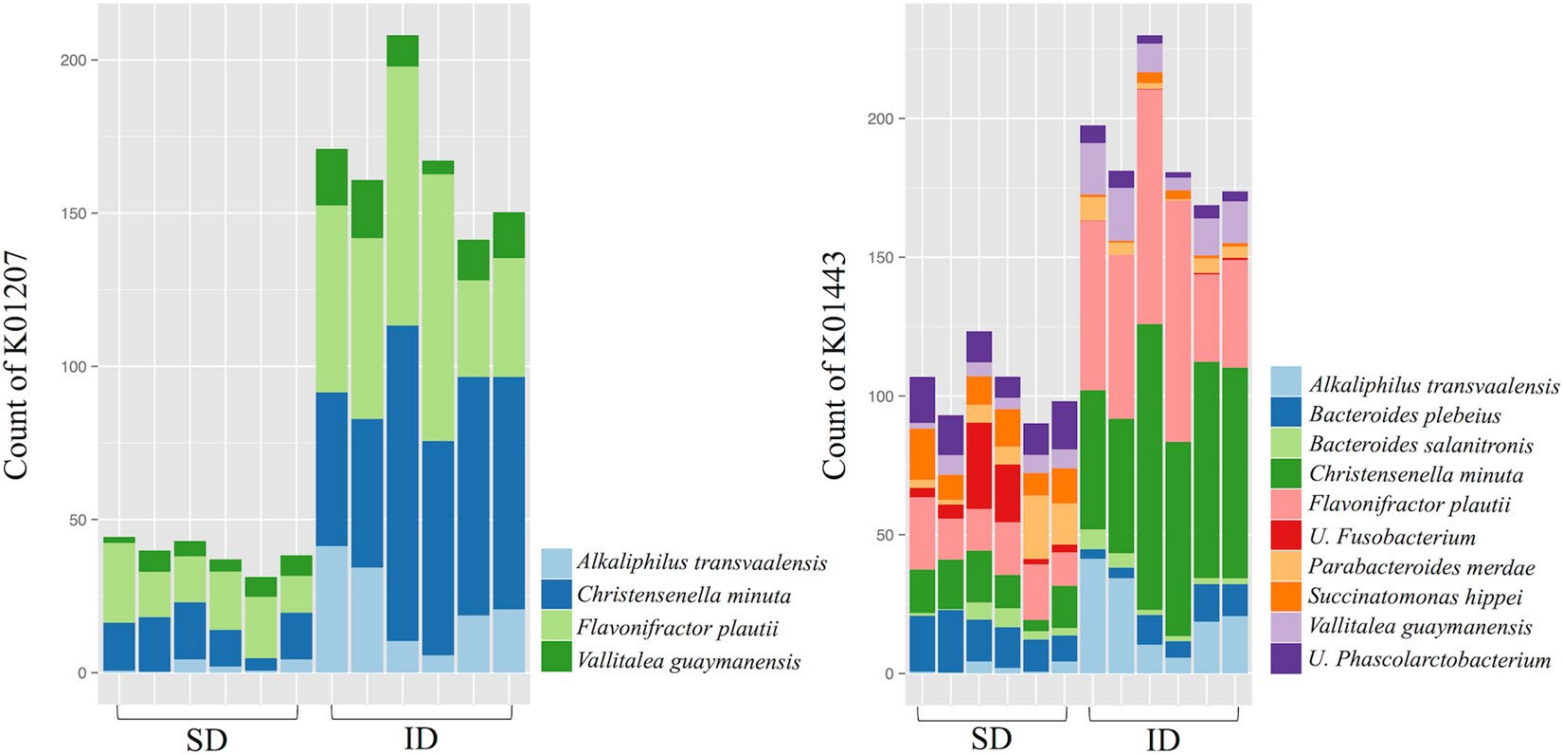
Stacked bar chart showing the count of metagenomic contributors for beta-N-acetylhexosaminidase (K01207) and N-acetylglucosamine-6-phosphate deacetylase (K01443) in SD and ID samples.

In order to translate the insect-diet fed gut microbiota in specific metabolic features of the corresponding microbiome, we applied Phylogenetic Investigation of Communities by Reconstitution of Unobserved State (PICRUSt) analysis^21^. K01183, K01207 and K01443 KEGG functions, involved in chitin metabolism, were studied in key bacteria discriminating ID and SD microbial communities to assess the connection between microbial chitin degradation and SCFAs production. PICRUSt analysis revealed low levels of bacterial chitinase (K01183) slightly enriched in SD group. Indeed, β-N-acetylhexosaminidases (K01207) and N-acetylglucosamine 6-phosphate deacetylase (K01443), the key enzymes that cleave oligomers produced by chitinase, were highly represented in microbial communities of both hen groups, showing an increased count in ID microbial communities (Table S2). The PICRUSt analysis reveals that principal contributors to β-N-Acetylhexosaminidase and N-acetylglucosamine 6-phosphate deacetylase abundance were *F*. *plautii*, *C*. *minuta* and *A*. *transvaalensis* (Fig. 3). Moreover KEGG orthologs were studied also for key enzymes involved in butyrate and propionate production from carbohydrates^22,23^. The majority of the functions analysed were enriched in ID group (Table S3).

### Cecal SCFAs concentration and correlation with key bacterial species

**Figure 4:**
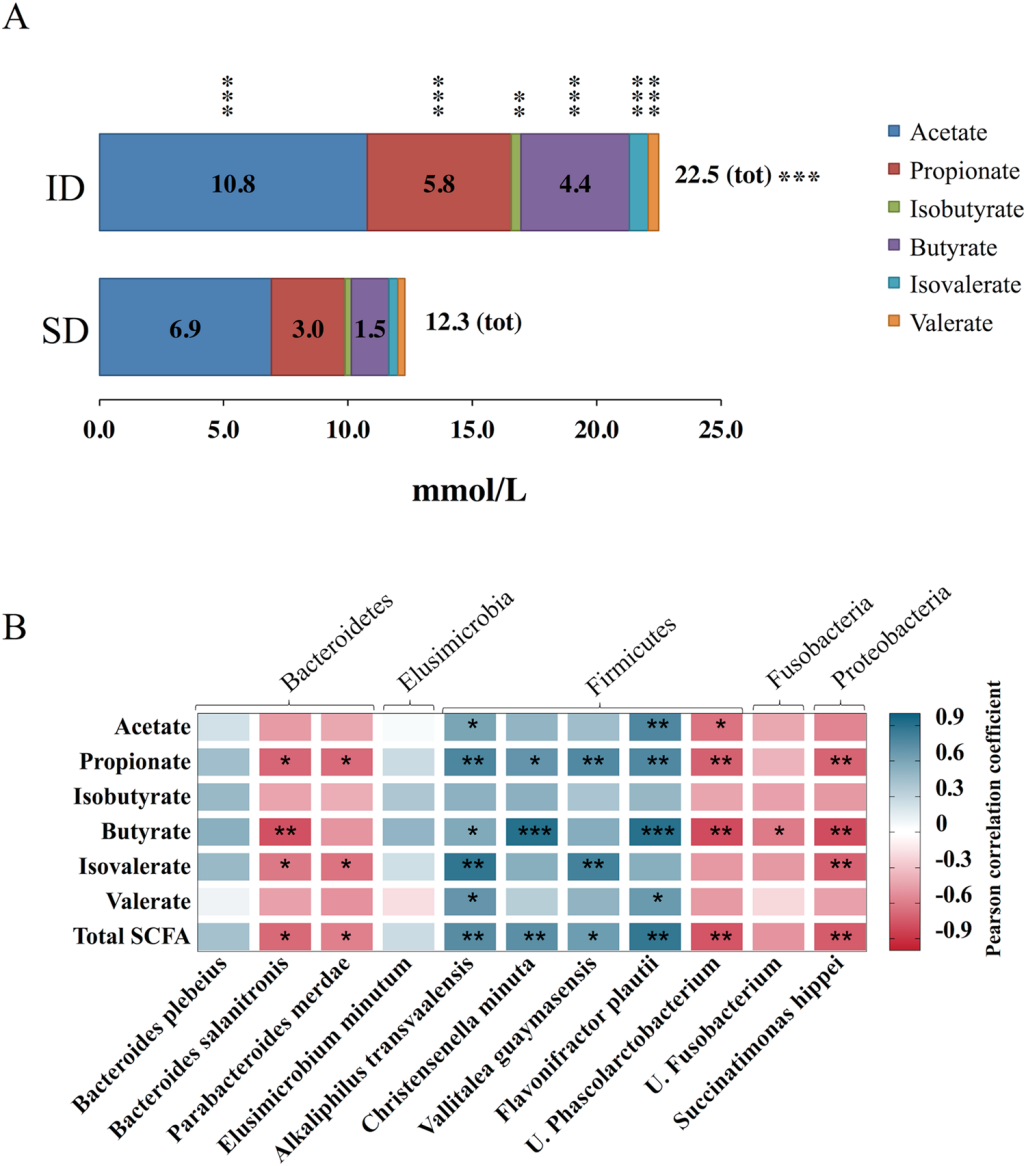
SCFAs in cecal samples of ID and SD groups. (**A**) Stacked bar chart shows mean values of cecal SCFAs in SD and ID groups. (**B**) Heatmap shows the Pearson’s correlation between SCFAs concentration (y-axis) and key genera at species level identified by SPINGO (bottom, x-axis). Blue and red colors designate, respectively, the positive and negative correlations. *p < 0.05, **p < 0.01, ***p < 0.001.

In order to functionally describe the observed reassembled gut microbiota resulting from ID administration, we investigated the SCFAs concentrations. The levels of acetate, propionate, isobutyrate, butyrate, isovalerate and valerate were measured in caeca by gas chromatography. ID administration significantly increased the production of all SCFAs analyzed with respect to SD (Fig. 4A). We used the Pearson correlation coefficient to associate the ID gut microbiota profile with the related SCFAs produced. High levels of *F*. *plautii* (key genus *Oscillospira*, according to Greengenes database), *C*. *minuta* and *A*. *transvaalensis* (key genus unclassified member of Clostridiales) strongly correlated with high production of propionate, butyrate and with total SCFAs. In contrast, *S*. *hippei* (class of Gammaproteobacteria) and *Phascolarctobacterium* (unclassified member of Veillonellaceae), correlated negatively with propionate, butyrate, isovalerate and with total SCFAs concentrations (Fig. 4B).

Principal component analysis (PCA) was applied to correlate the relative amount of each key variable in all samples (significant species, SCFAs levels, body weight, feed intake, cholesterol and triglycerides serum concentrations). We found a strong clustering of ID away from SD groups, indicating that diet may drive all variables considered (Fig. S2).

## Discussion

The present study represents a comprehensive evaluation of microbial communities modulation and SCFAs production after insect larvae meal administration in laying hens.

*H*. *illucens* had no negative effects on laying hen health status. Lower serum cholesterol and triglycerides levels and a reduction of feed intake and body weight were observed in the ID group with respect to SD group, as also reported by Marono *et al*. (2017)^18^. Moreover, ID group showed a lower cholesterol content in the egg yolks produced (Table S1) according to Noguiera *et al*. (2003)^24^ where diet containing up to 30 g/kg chitosan reduced cholesterol egg yolk contents. In our study the inclusion of *H*. *illucens* larvae in hen diet, was shown to have an important effect in modulating the gut microbiota communities and their metabolites. Statistical analysis showed that the composition of the microbial community correlated significantly with SCFAs levels measured in the caeca during the different dietary administration.

Overall, the new microbiota detected in ID group showed a higher microbial richness compared to SD group. The acquired microbial richness, in the symbiotic relationship with the host, may potentially provide further metabolic capabilities. Several studies, in fact, revealed that a rich bacterial community is associated with a healthy status, while a lower bacterial diversity, with reduction of metabolic, immunological and gut protective functions, is related to several diseases^25^. Moreover, comparing the ID and SD bacterial communities by beta-diversity analysis, the main differences within the two groups were due to a different assortment in bacterial species (Fig. 1B). The new equilibrium in gut microbiota of the ID group, with respect to SD group, was characterized by the significant increase of *Bacteroides plebeius*, *Elusimicrobium minutum*, *Alkaliphilus transvaalensis*, *Christensenella minuta*, *Vallitalea guaymasensis* and *Flavonifractor plautii*. Consequently, these bacteria represent the major contributors to changes in gut microbiota composition of ID group. Furthermore, a different assortment in bacterial species belonging to the order of Clostridiales was observed. In particular, within this order, *F*. *plautii* (genus *Oscillospira*) and *C*. *minuta* were already reported in metagenomic studies as highly positively associated each with other, possibly in a mutualistic relationship^26^. These bacteria are currently under scrutiny, raising particular interest since their abundance was found to correlate with the lean host phenotype^27,28^. Moreover, *Oscillospira* is the only genus that increased also in the caecum during fasting in birds, fishes and mammals^29^. These evidences matched with the reduced body weight and the increase of *Oscillospira* in ID group.

The increase of *Oscillospira* after this specific diet administration in laying hens is of considerable interest. *Oscillospira* is actually an enigmatic bacterial genus, butyrate producer that may contribute to the formation of secondary bile acids and known to prevent *Clostridium difficile-*associated infectious disease (CDAD) in humans^26,30,31^. Poultry represents a possible reservoir of *C*. *difficile* ^32^ and our study suggests that *H*. *illucens* larvae administration may have a potential antimicrobial effect against *C*. *difficile* infection, by increasing the genus *Oscillospira*.

The insect meal administration may affect also the levels of SCFAs, end products of bacterial fermentation. SCFAs, such as butyric, propionic and acetic acids, once absorbed into the blood, bio-regulate and promote mucosal growth via direct or indirect mechanisms in the gut, as well influencing metabolism systemically^33^. SCFAs play an important role in gut physiology, and especially butyrate, serves as energy source for colonic epithelial cells. In addition, SCFAs negatively affect the expression of virulence factors of bacterial pathogens^13^. In our study, total SCFAs, in particular propionate and butyrate, were higher in ID than in SD hens. This increase in SCFAs concentrations may be the result of modulation in the microbial population induced by the insect-based diet. Moreover, these bacterial metabolites also contribute to mineral uptake and provide extra energy to the birds^34^, adjusting, in turn, the intestinal microbiota ecosystem towards a host-friendly environment^35^. This effect may preserve the maintenance of the described new equilibrium induced by *H*. *illucens* larvae diet administration.

Also on the basis of our results, *H*. *illucens* larvae dietary administration may act as a potential prebiotic and chitin may be the key feature of this effect. The chitin content in the insect meal might serve as substrate for the gut microbiota, influencing the composition and the microbial fermentation metabolites. Chitin is the second most abundant polysaccharides in nature of β-1, 4-linked *N*-acetyl-D-glucosamine (GlcNAc). Chicken has the acidic chitinase in stomach and in the intestine that breaks down chitin-containing insect in dimers of GlcNAc to produce chitooligosaccarides^36^. PICRUSt analysis revealed low levels of bacterial chitinase (K01183), slightly enriched in SD group. Interestingly, more than 4–fold increase of β-N-acetylhexosaminidases (K01207) was observed in ID microbial communities. β-N-acetylhexosaminidases (K01207) is the second main class of chitinolytic enzymes that degrade chitooligosaccharides formed by chitinases into monomers^37^. This enzyme is also attracting particular interest for oligosaccharide synthesis, but the sources of this enzyme are rare^38^ and the insect-diet fed bacteria might represent an important source of this exogenous chitinase. GlcNAc is deacetylated by *N*-acetylglucosamine 6-phosphate deacetylase (K01443), an enzymatic function increased in ID group, to glucosamine-6-phosphate and acetate^39^. Glucosamine-6-phosphate and acetate can be co-metabolized by cross-feeders bacteria to biosynthesize SCFAs^40–42^. Therefore, K01207 and K01443 enriched functions in microbial communities of ID group represent the key enzymes responsible for the degradation of chitin derivatives from chicken chitinase and higher SCFAs production. Of note, the principal contributors to K01207 and K01443 abundance were *F*. *plautii* (key genus *Oscillospira*), *C*. *minuta* and *A*. *transvaalensis*. Interestingly, we found a strong correlation between levels of *F*. *plautii*, *C*. *minuta* and *A*. *transvaalensis* with high production of propionate, butyrate and total SCFAs. Genus *Alkaliphilus*, isolated in soil and also in chicken, dismutates crotonate to acetate and butyrate and its relative abundance is influenced by diets^43–45^. Moreover, PICRUSt analysis for key enzymes involved in butyrate and propionate production revealed an enrichment of several KEGG orthologs in ID group (Table S3). Thus, considering the beneficial metabolic features of SCFAs, these bacteria, also involved in chitin degradation, may contribute to hen health during insect diet such as lowering blood triglycerides and cholesterol levels.

The reduced feed intake during insect meal administration reported by Marono *et al*. (2017)^18^ was probably promoted by increased of satiety. It has been suggested that SCFAs, derived from bacterial fermentation of dietary fibers, stimulate the production and secretion of satiety-related hormones associated with fed or fasted state (GLP-1 and PYY)^46^. In our study taxa of *Oscillospira* genus and *C*. *minuta*, highly correlated with production of butyrate and total SCFAs. These evidences matched with the reduced feed intake and consequently body weight registered in ID group. This effect is supported by the reported association of *Oscillospira* and *Christensenella* with lean host phenotype^27,28^. Furthermore, the insect-meal administration also increased the levels of propionate, produced as a result of fermentative activity of gut microbiota during fiber degradation. Propionate can penetrate the blood–brain barrier, produce widespread effects on neurotransmitters release (dopamine and serotonin) and affects mitochondrial fatty acid metabolism^47^. Recent findings from human and rodent studies suggest that propionate production may play an important role in attenuating reward-based eating behavior via striatal pathways, independently of changes in plasma satiety-related hormones^48^. On the other hand, it has also been suggested that propionate regulates appetite by stimulating GLP-1 and PYY secretion^49^. Finally, SCFAs serve as an immediate energy source when glucose absorption is decreasing in the small intestine, stabilizing glucose levels in blood^50^.

We hypothesized that insect-based diet and chitin content may have positive metabolic health effects, such as increasing satiety, lowering blood triglycerides and cholesterol levels. These effects may be associated with SCFAs derived by microbial degradation of insect meal, as also described in *in vitro* and *ex vivo* previous studies^51,52^. Our data strongly corroborate the hypothesis of possible beneficial effects of insect-based diet on global health of hens, even though further studies are necessary to decipher its precise impact on human and animal physiology.

## Conclusion

Changes in the gut microbiota as a result of feeding with insect larvae meal are novel and highly significant in our study. Dietary substances like chitin, one of the principal biopolymer in insect meal, plus bacteria in the gut, may work together to produce SCFA molecules. Here the microbiota plays a key role in the coordination of polysaccharide degradation responsible for the increases in SCFAs concentrations, promoting both gut and overall health. Considering that insects are already part of the human and animal diet in many countries, their potential as nutritional source needs to be re-evaluated. Insects can partly replace the protein ingredients in the livestock and poultry industries. For this reason the aim for 2020 is to introduce farmed insects as ingredients for feed and food^1^. Fermentable chitin, as a potential prebiotic, may determine a healthy and a well-fed microbiota in laying hens. How do the gut microbiota exerts its effect in the intestinal tract and throughout the rest of the body is actually a crucial question.

We believe that the beneficial effects on hens during insect diet administration, supported by here reported results, deserve further investigation, especially considering the current lack of knowledge and the growing worldwide interest in insect-based food for animals and humans.

## Materials and Methods

### Ethic Statement

All hens were treated in accordance will Directive of the European Parliament of the Council on the Protection of Animals Used for Scientific Purpose and in agreement with the Institutional Animal Care and Use Committee of the University of Naples Federico II, D.lgs n. 26 04/03/2014. All experiments involving hens were approved by the Bioethical Committee of the University of Naples Federico II, under number of protocol: 2017/0017676.

### Birds and Experimental Design

A total of 24 Lohmann Brown Classic laying hens, representing a subgroup of those employed in a previous study^18^ and obtained from a commercial hatchery, were randomly and equally divided in two different diet group (12 hens per group), each divided into 3 replicates of 4 hens each. The experiment was conducted on hens from 24 to 45 weeks old, housed in modified cages (800 cm^2^/hen), under controlled temperature and humidity conditions. The hens, visually inspected daily, had free access to feed and fresh water, that were daily distributed; the dark:light cycle was 9:15 hours. The experimental cages were surrounded by commercial hens flocks composed of birds of the same origin as those used in the experiments. The cages had the same dimensions and the same number of drinkers and feed hoppers to simulate commercial production conditions and three hens per cage were housed for each group.

### Diets and Feeding Program

The two groups were fed *ad libitum* with isoproteic and isoenergetic diets: Soybean meal (SD) group was fed a corn-soybean-based diet, while in the *H*. *illucens* larvae meal (ID) group, the soybean was completely replaced by a defatted meal of *H*. *illucens* larvae (Hermetia Deutschland GmbH & Co KG, Amtsgericht Potsdam, DE). The diets were formulated according to Lohmann Brown classic Management Guides (2013)^53^. Chemical characteristics of the two different diets (insect meal and soybean meal), including the amount of chitin, are as reported by Marono *et al*. (2017)^18^. In particular, taking into account the average feed intake and the level of inclusion of the insect meal in the diet, the hens from ID group ingested around 1.02 g/d of chitin all along the trial.

### Data and sample collection

Feed intake and body weight of birds were measured once a week throughout the entire experiment as reported by Marono *et al*., (2017)^18^ (Table 1). At the end of the trial (45 weeks of age), all hens were euthanized by cervical dislocation and dissected under sterile condition. Two hens per replicate (6 per group) were analyzed. From each carcass the caeca were tied at both ends, separated by sterile instruments from the rest of the gastrointestinal tract, placed in a sterile 15 ml falcon and stored at −80 °C. Each samples collected contained two caeca belonging to 2 birds of the same experiment, for a total of 12 samples pooled of approx. 5 g each. One caecum was used for microbial communities identification and the other one for SCFA analysis.

### Microbial DNA extraction and 16S rDNA sequencing

Bacterial genomic DNA was extracted from caeca of each pool using the QIAamp DNA Stool Mini Kit (Qiagen) according to manufacturer’s instructions. DNA concentrations were measured fluorometrically using the Qubit dsDNA BR assay kit (Invitrogen) and quality was assessed by spectrophotometric measurements with NanoDrop (ThermoFisher Scientific Inc.). DNA samples were stored at −20 °C until processed for amplification.

Sequencing samples were prepared according to the protocol 16S Metagenomic Sequencing Library Preparation for Illumina Miseq System, with some modifications. The V3-V4 regions of the 16S rDNA gene were amplified starting from 200 ng of DNA template in a reaction volume of 50 µL containg 1x Fast start High Fidelity Reaction Buffer, 5 μM of each primer, 0.2 nM of dNTPs, 3 mM MgCl_2_, and 2 U FastStart High Fidelity PCR System (Roche Applied Science). PCR was performed using the following cycles conditions: an initial denaturation step at 95 °C for 2 min, followed by 30 cycles of 95 °C for 30 s, 55 °C for 45 s, 72 °C for 55 s and ended with an extension step at 72 °C for 5 minutes; products were visualized by electrophoresis on 1.2% agarose gel. After a purification step with Agencourt AMPure XP (Beckman Coulter Inc), the amplicons were indexed with 10 subsequent cycles of PCR using the Nextera XT Index Kit (Illumina). The second PCR was performed according to Illumina guidelines using the KAPA HiFi HotStart System. Amplicons were visualized using gel electrophoresis and subsequently cleaned as described above. Library sizes were assessed using a Bioanalyzer DNA 1000 chip (Agilent technologies) and quantified by Qubit. Normalized libraries were pooled, denatured with NaOH, then diluted to 10 pM and combined with 25% (v/v) denatured 10pM PhiX, according to Illumina guidelines. Sequencing run was performed on an Illumina Miseq system using v3 reangents for 2 × 281 cycles.

### Metagenomic data analysis

V3-V4 16S rDNA FASTQ paired-end reads were pre-processed with PEAR^54^ in order to assemble reads with an overlap of at least 40 nucleotides, and to retain high quality sequences (PHRED score ≥ 33) that were comprised between 400 and 500 bp. Filtered sequences were then processed with PRINSEQ^55^ in order to obtain FASTA and quality files for further analyses. Metagenomic analyses on the resulting data were conducted using Quantitative Insights Into Microbial Ecology (QIIME, version 1.8.0)^56^. 16S sequences were used to pick OTUs at 97% of sequences similarity from Greengenes 16S gene database (GG, may 2013 version)^57^ with a closed reference-based OTU picking method. The GG database was used to taxonomically classify the identified OTUs and to compute their distribution across different taxonomic levels. Species and Clostridium cluster classification was performed using SPecies level IdentificatioN of metaGenOmic amplicons program (SPINGO version 1.3) with default parameters on a representative sequence of each OTU^20^. To avoid sample size biases in subsequent analyses, a sequence rarefaction procedure was applied using a maximum depth of 5,234 sequences/sample. To assess sampling depth coverage and species heterogeneity in each sample, alpha diversity metrics were employed on rarefied OTU table using Good’s coverage, Observed species and Shannon’s diversity indexes. A two-sample permutation t-test, using 999 Monte Carlo permutations to compute p-value, was performed to compare alpha diversities between sample groups. OTUs diversity among sample communities (beta diversity) was assessed by applying unweighted and weighted Unifrac distances and compared using the ANOSIM method^58^ with 999 permutations. Statistical differences in OTUs frequencies between SD and ID groups at different taxonomic levels were assessed using nonparametric Kruskal-Wallis test, with False discovery rate (FDR) correction for multiple testing. Finally, unweighted unifract distances and species level classification data were visualized by principal coordinate analysis (PCoA) biplots including the 10 most abundant bacterial species driving the samples clustering. Metagenomes of key species were predicted using PICRUSt based on normalized OTU table, corrected for multiple 16S rRNA gene copy number^21^. Kyoto encyclopedia of genes and genomes^59^ ortholog abundances of  chitinase [EC:3.2.1.14] (K01183), beta-N-acetylhexosaminidase [EC:3.2.1.52] (K01207) and N-acetylglucosamine-6-phosphate deacetylase [EC:3.5.1.25] (K01443) between groups were compared using two tailed Student’s t-test. Predicted contribution of each key bacterial species to K01207 and K01443 counts were computed using the script metagenome_contributions.py in PICRUSt and visualized with an Rscript in Microbiome Helper^60^.

### Analysis of SCFAs production

Cecal digesta samples (each about 5 ml) after dilution with oxalic acid (1:1, v/v), were subjected to short chain fatty acids (SCFA) analysis by gas chromatography (Thermo-Electron mod. 8000top, FUSED SILICA Gaschromatograph (ThermoElectron Corporation, Rodano, Milan, Italy) with OMEGAWAX 250 fused silica capillary column 30 m × 0.25 mm × 0.25 mm film thickness; analysis temperature, 125 °C; flame ionisation detector, 185 °C; carrier helium, 1.7 ml/min (Stanco *et al*., 2003).

### Other statistical methods

Cecal SCFAs concentrations in SD and ID samples were compared using two tailed Student’s t-test assuming equal variance. Pearson correlation test was used to assess the eventual association between the amount of key bacterial species and SCFAs levels. Principal component analysis (PCA) was performed on the abundance of each key variable/sample (significant bacterial species, SCFAs levels, body weight, feed intake, cholesterol and triglycerides concentrations) using JMP software (SAS, Cary, NC).

In this study results were considered statistically significant at p-value < 0.05. Significant differences were indicated in figures and tables by *p < 0.05, **p < 0.01, ***p < 0.001. Bar plots were created by using GraphPad Prism 6.0.

## Electronic supplementary material

Supplementary information
