# Supplementary material for: Insect-based diet, a promising nutritional source, modulates gut microbiota composition and SCFAs production in laying hens
Source: Sci Rep. 2017 Nov 24;7:16269. doi: 10.1038/s41598-017-16560-6 (PMC5701250; doi:10.1038/s41598-017-16560-6)

Insect-based diet, a promising nutritional source, modulates gut microbiota composition and SCFAs production in laying hens

Luca Borrelli[1,6,*,+], Lorena Coretti[2,3,6,+], Ludovico Dipineto[1,6], Fulvia Bovera[1], Francesca Menna[1,6], Lorenzo Chiariotti[2,4,6], Antonio Nizza[1], Francesca Lembo[5,6] and Alessandro Fioretti[1,6]

[1]Department of Veterinary Medicine and Animal Productions, University of Naples Federico II, Naples, Italy

[2]Institute of Experimental Endocrinology and Oncology "Gaetano Salvatore" IEOS, National Research Council CNR, Naples, Italy

[3]Department of Advanced Biomedical Science, University of Naples Federico II, Naples, Italy.

[4]Department of Molecular Medicine and Medical Biotechnologies, University of Naples Federico II, Naples, Italy

[5]Department of Pharmacy, University of Naples Federico II, Naples, Italy

[6]Task Force on Microbiome Studies, University of Naples Federico II, Naples, Italy.

*luca.borrelli@unina.it

[+]These authors contributed equally to this work.

Supplementary information

Supplementary Table 1. Total lipids and cholesterol determination in egg yolks.

Parameter	SD group	ID group
Total lipids, g/100 g	32.60 ± 0.72	30.93 ± 0.83
Cholesterol, mg/100 g lipids	0.386 ± 0.002	0.312 ± 0.004*

Total lipids, measured according to Folch et al (1957)[59] and cholesterol levels measured by gas-chromatograph method[60] of twenty-one eggs for each group collected during eight different deposition days (means ± standard errors). P-values were calculated by two tailed Student's t-test; *p<0.05.

Supplementary Table 2. KEGG Orthologs count of chitinase (K01183), beta-N-acetylhexosaminidase (K01207) and N-acetylglucosamine-6-phosphate deacetylase (K01443) in SD and ID groups.

KEGG Orthologs	KEGG Description	SD group	ID group
K01183	chitinase [EC:3.2.1.14]	14.50 ± 1.20	9.17 ± 0.7**
K01207	beta-N-acetylhexosaminidase [EC:3.2.1.52]	38.83 ± 1.92	166.33 ± 9.48***
K01443	N-acetylglucosamine-6-phosphate deacetylase [EC:3.5.1.25]	103.00 ± 4.92	188.83 ± 9.16***

Data are means and standard errors. P-values were calculated by two tailed Student's t-test (**p<0.01, ***p<0.001).

Supplementary Table 3. KEGG Orthologs count of genes codifying for key enzymes involved in butyrate and propionate production from carbohydrates in SD and ID groups.

KEGG Orthologs	KEGG Description	SD group	ID group
		**PIRUVATE ➔ BUTYRATE**	
K00170/1/2	pyruvate ferredoxin oxidoreductase, beta/ delta/gamma subunit [EC:1.2.7.1]	209.84 ± 58.09	364.84 ± 78.44
K00626	acetyl-CoA C-acetyltransferase [EC:2.3.1.9]	119.34 ± 39.24	125 ± 14.9
K00074	3-hydroxybutyryl-CoA dehydrogenase [EC:1.1.1.157]	101.5 ± 21.06	228.84 ± 18.07***
K01692	enoyl-CoA hydratase [EC:4.2.1.17]	36.34 ± 2.3	64.84 ± 8.91*
K00248	butyryl-CoA dehydrogenase [EC:1.3.8.1]; butyryl-CoA dehydrogenase [EC:1.3.99.2]	143.34 ± 42.64	209.67 ± 26.62
K00634	phosphate butyryltransferase [EC:2.3.1.19]	90.34 ± 20.6	108.17 ± 12.29
K00929	butyrate kinase [EC:2.7.2.7]	90.34 ± 20.6	108.17 ± 12.29
		**PIRUVATE ➔ ACETATE**	
K04020	phosphotransacetylase	0	0
K00925	acetate kinase [EC:2.7.2.1]	138 ± 20.64	264.34 ± 24.46**
		**ACETATE ➔ BUTYRATE**	
K01034	acetate CoA-transferase alpha subunit [EC:2.8.3.8]	57.34 ± 17.96	62 ± 9.76
		**PIRUVATE ➔ SUCCINATE**	
K01958	pyruvate carboxylase [EC:6.4.1.1]	66.17 ± 18.91	100.34 ± 13.99
K00024	malate dehydrogenase [EC:1.1.1.37]	64.17 ± 3.05	25.5 ± 1.03***
K01677	fumarate hydratase subunit alpha [EC:4.2.1.2]	109 ± 19.74	251.67 ± 24.47***
K01678	fumarate hydratase subunit beta [EC:4.2.1.2]	100.84 ± 20.07	229.17 ± 25.16**
K00239	succinate dehydrogenase flavoprotein subunit [EC:1.3.99.1]	89.5 ± 20.25	27.34 ± 2.65*
		**SUCCINATE ➔ BUTYRATE**	
K01903	succinyl-CoA synthetase beta subunit [EC:6.2.1.5]	25.17 ± 2.47	6.34 ± 0.5***
K01902	succinyl-CoA synthetase alpha subunit [EC:6.2.1.5]	25.17 ± 2.47	6.34 ± 0.5***
K15038	succinyl-coA reductase [EC:1.2.1.76]	0	0
K00043	4-hydroxybutyrate dehydrogenase [EC:1.1.1.61]	0	0
K14472	succinyl-CoA:(S)- malate CoA transferase subunit B [EC:2.8.3.-]	0	0
K14471	succinyl-CoA:(S)- malate CoA transferase subunit A [EC:2.8.3.-]	0	0
K14534	4-hydroxybutyryl-CoA dehydratase / vinylacetyl-CoA-Delta-isomerase [EC:4.2.1.120 5.3.3.3]	38.5 ± 1.41	138.17 ± 20.66***
		**SUCCINATE ➔ PROPIONATE**	
K01903	succinyl-CoA synthetase beta subunit [EC:6.2.1.5]	25.17 ± 2.47	6.34 ± 0.5***
K01902	succinyl-CoA synthetase alpha subunit [EC:6.2.1.5]	25.17 ± 2.47	6.34 ± 0.5***
K01847	methylmalonyl-CoA mutase [EC:5.4.99.2]	86.34 ± 4.73	39.17 ± 2.14***
K05606	methylmalonyl-CoA epimerase [EC:5.1.99.1]	0 ± 0	0 ± 0
K03416	methylmalonyl-CoA carboxyltransferase [EC:2.1.3.1]	0 ± 0	0 ± 0
		**GLUCOSE ➔ PROPIONATE**	
K00016	L-lactate dehydrogenase [EC:1.1.1.27]	65.17 ± 4.24	175.17 ± 9.11***
K01026	propionate CoA-transferase [EC:2.8.3.1]	62.67 ± 20.3	62.34 ± 9.68

Data are means and standard errors. P-values were calculated by two tailed Student's t-test (*p<0.05, **p<0.01, ***p<0.001).

Supplementary figure 1. Unweighted PCoA biplot displays ten more abundance species (grey spheres) that drive the samples clustering (5,234 sequences/sample). The size and location of the grey spheres are plotted according to presence/absence of the respective OTUs in each sample.

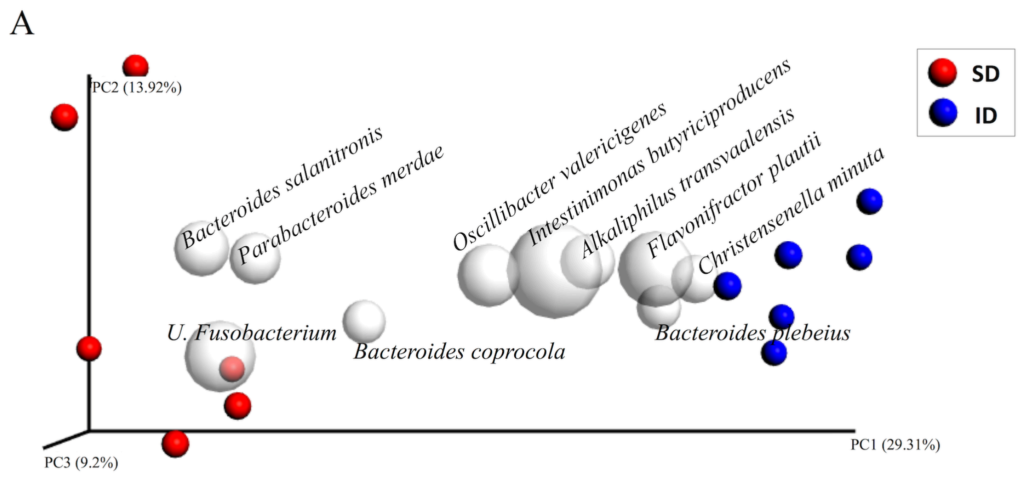

Supplementary figure 2. PCA of significant bacterial species, SCFAs levels, body weight, feed intake, cholesterol and triglycerides concentrations distribution in ID and SD samples. The vectors represented by grey arrows show how (the direction) and how much (the length) each parameter contributes to the total correlations represented by PC1 and PC2.

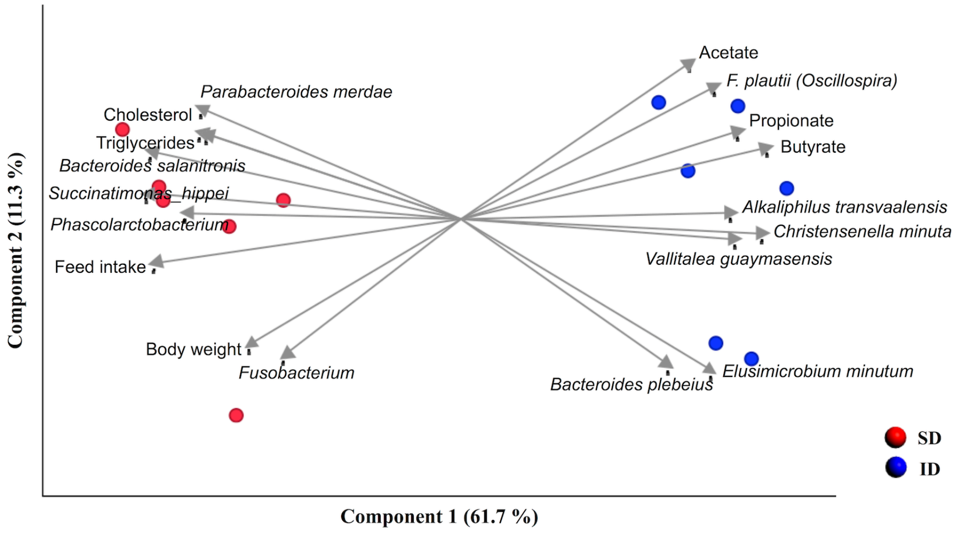

Supplement: Supplementary file 1 — Supplementary information [file 41598_2017_16560_MOESM1_ESM.pdf]
